# Supplementary material for: Mueller Matrix Polarimetry on Cyanine Dye J-Aggregates
Source: Molecules. 2023 Feb 4;28(4):1523. doi: 10.3390/molecules28041523 (PMC9960244; doi:10.3390/molecules28041523)
Supplement: Supplementary file 1 [file molecules-28-01523-s001.zip › molecules-2164537-supplementary.pdf]

Supplementary Materials for

Mueller Matrix Polarimetry on cyanine dye J-aggregates

Samuel R. Clowes[1], Dora M. Răsădean[1], Tiberiu-M. Gianga[2], Tamás Jávorfi[2], Rohanah Hussain[2], Giuliano Siligardi[2], and G. Dan Pantoş[1], *

[1]Department of Chemistry, University of Bath, Claverton Down, Bath, BA2 7AY, UK;

[2]B23 beamline, Diamond Light Source, Didcot, OX11 0DE, UK;

*Correspondence: g.d.pantos@bath.ac.uk

1. CD plots from benchtop spectrometers 1
2. CD plots from SRCD 2
3. Individual Mueller Plots 5
4. Combined Normalised via Z-scores Mueller Plots 10
5. Mueller mapping plots of J-aggregate Films 11

1. CD plots from benchtop spectrometers

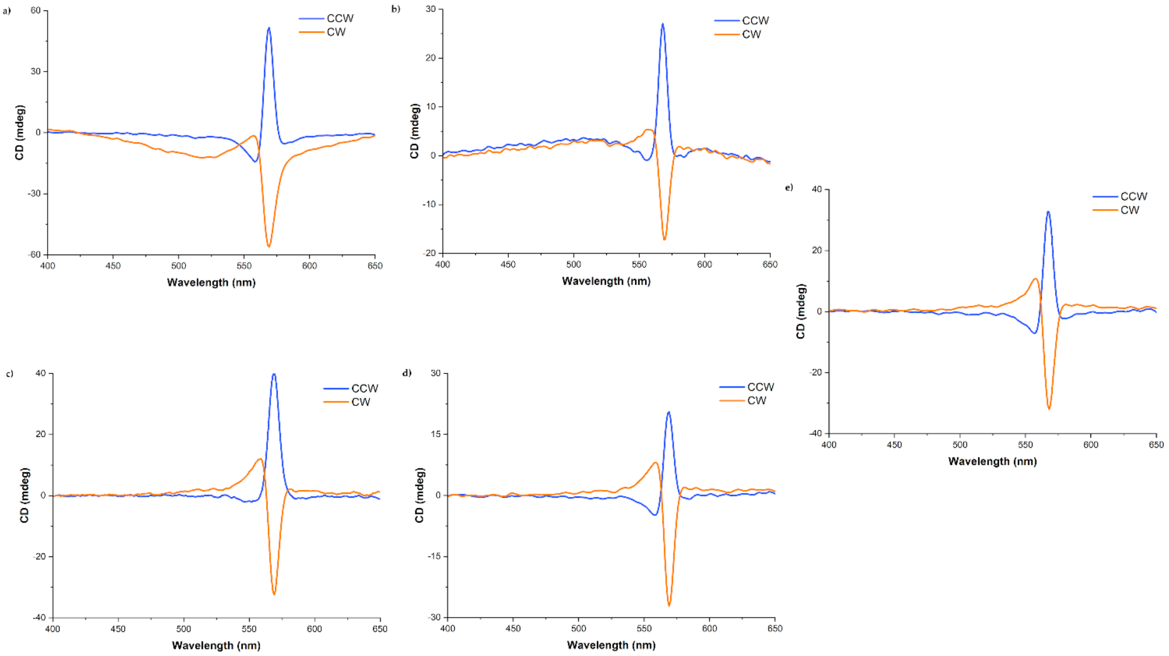

Figure S1: The CD plots from solutions of S0271 in Milli-Q water (pH 7.12, 18.2 Ω, recorded at 21 °C), stirred CW (orange) and CCW (blue) at **a**) 15 μM, **b**) 25 μM, **c**) 35 μM, **d**) 50 μM, and **e**) 75 μM.

2. CD plots from SRCD

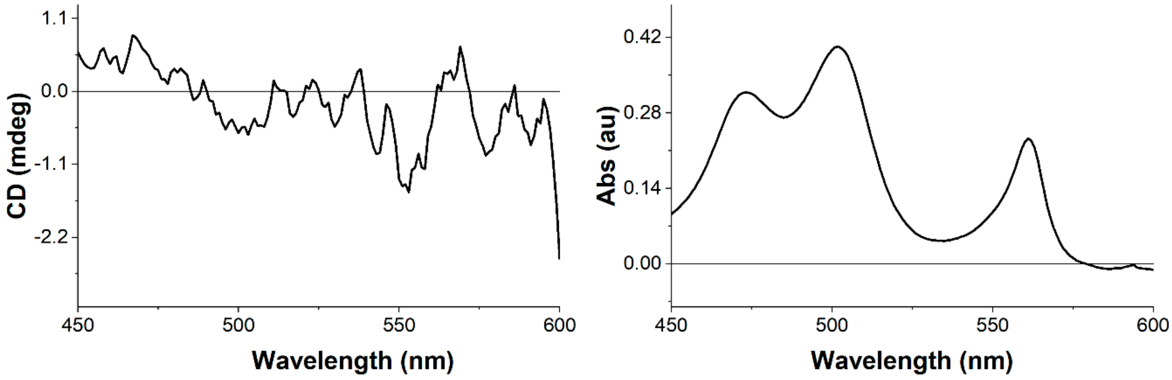

Figure S2: CD and UV-vis absorbance for a non-stirred solution of S0271 at 25 μM in Milli-Q water (pH 7.12, 18.2 Ω, recorded at 24 °C).

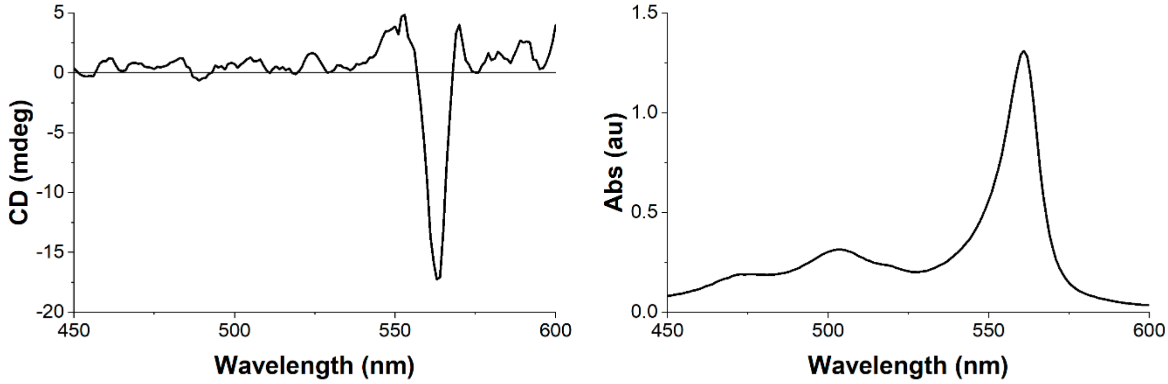

Figure S3: CD and UV-vis absorbance for a solution of S0271 at 25 μM stirred clockwise in Milli-Q water (pH 7.12, 18.2 Ω, recorded at 24 °C).

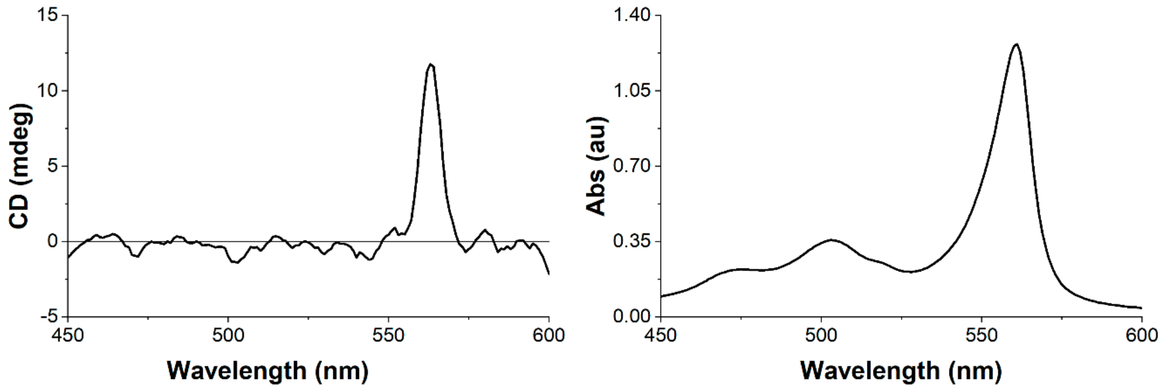

Figure S4: CD and UV-vis absorbance for a solution of S0271 at 25 μM stirred counterclockwise in Milli-Q water (pH 7.12, 18.2 Ω, recorded at 24 °C).

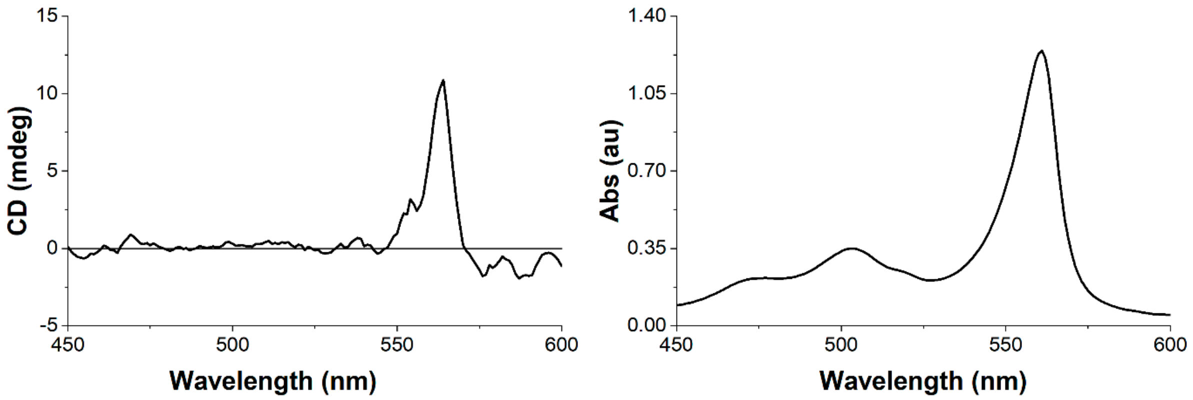

Figure S5: CD and UV-Vis absorbance for a solution of S0271 at 25 μM stirred counterclockwise in Milli-Q water (pH 7.12, 18.2 Ω, recorded at 24 °C). The cuvette was inverted relative to that for **Figure S4**.

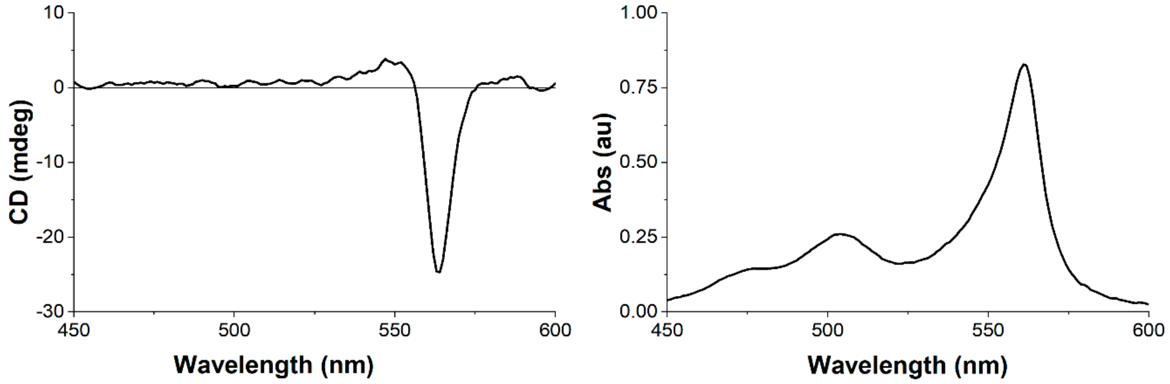

Figure S6: CD and UV-vis absorbance for a solution of S0271 at 15 μM stirred clockwise in Milli-Q water (pH 7.12, 18.2 Ω, recorded at 24 °C).

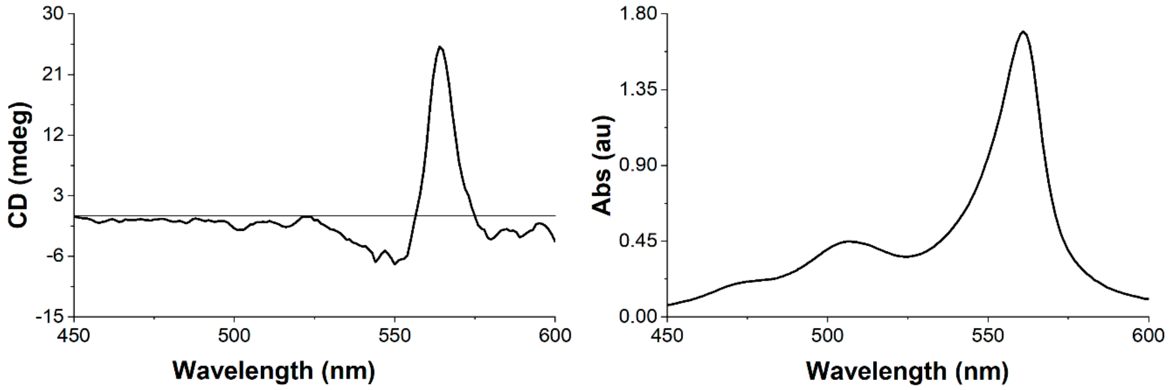

Figure S7: CD and UV-vis absorbance for a solution of S0271 at 15 μM stirred counterclockwise in Milli-Q water (pH 7.12, 18.2 Ω, recorded at 24 °C).

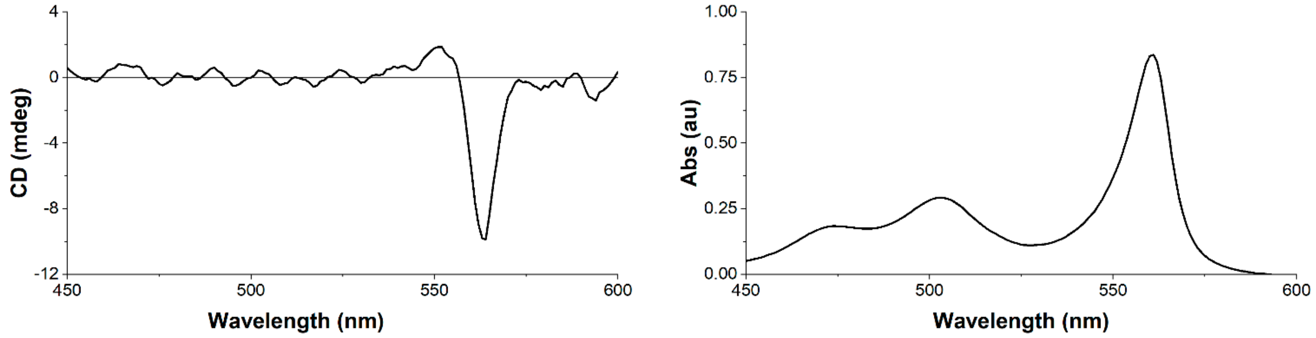

Figure S8: CD and UV-vis absorbance for a solution of S0271 at 35 μM stirred clockwise in Milli-Q water (pH 7.12, 18.2 Ω, recorded at 24 °C).

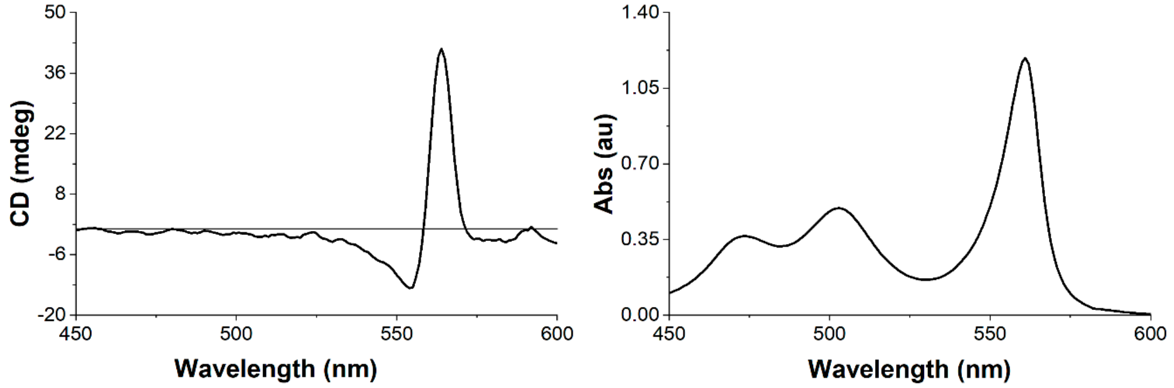

Figure S9: CD and UV-vis absorbance for a solution of S0271 at 35 μM stirred counterclockwise in Milli-Q water (pH 7.12, 18.2 Ω, recorded at 24 °C).

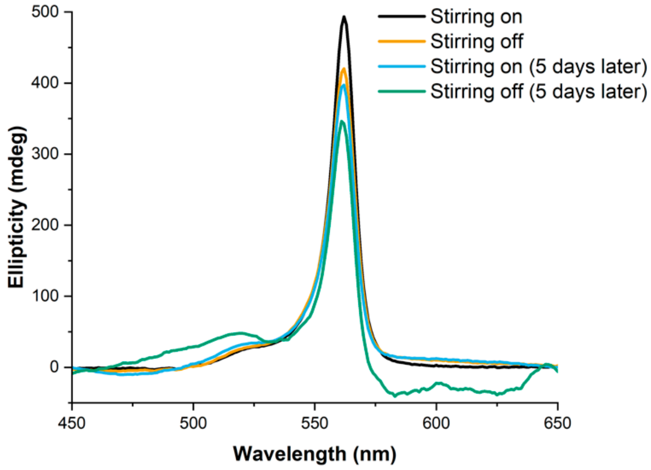

Figure S10: Comparison of CD spectra of S0271 J-aggregates while stirring, immediately after stirring was turned off and the same solution after being left for 5 days either with or without stirring.

3. Individual Mueller Plots

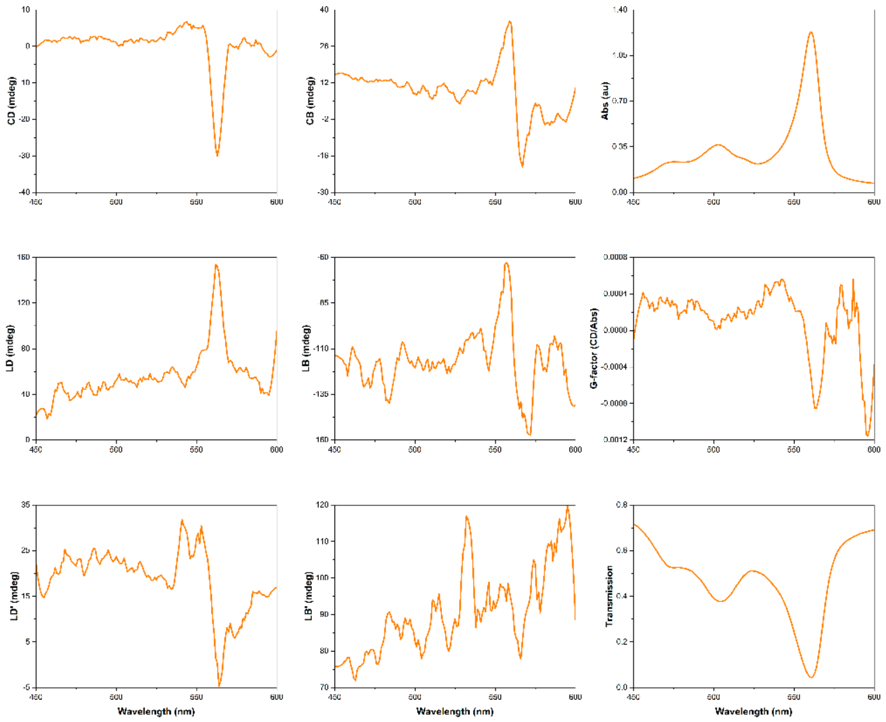

Figure S11: J-aggregates scanned on the MMP formed from stirring a 25 μM solution of S0271 clockwise in Milli-Q water (pH 7.12, 18.2 Ω, recorded at 24 °C). The data shown in this figure has been averaged from 4 scans and smoothed.

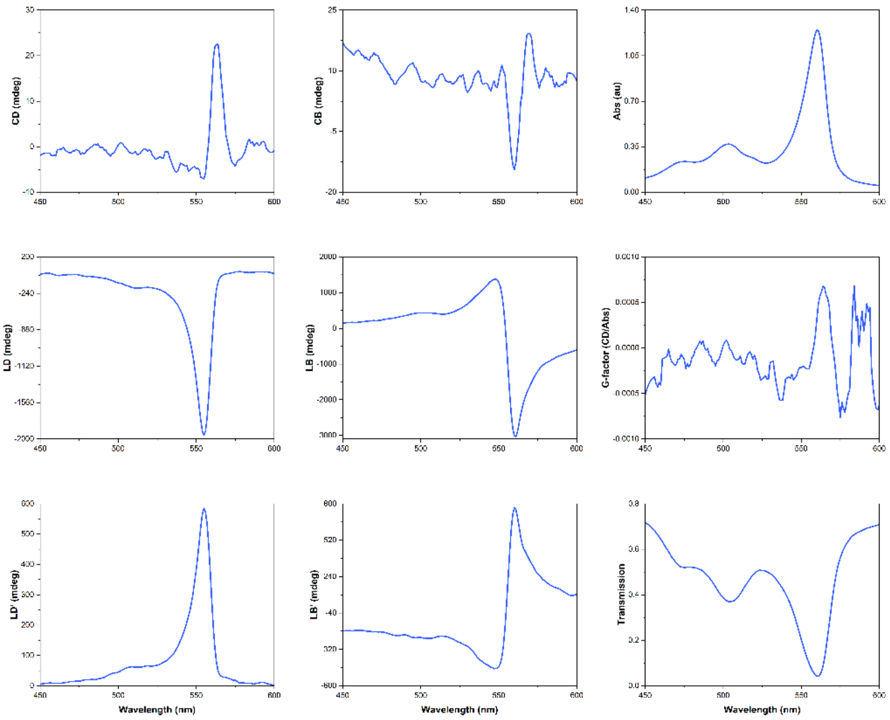

Figure S12: J-aggregates scanned on the MMP formed from stirred a 25 μM solution of S0271 counterclockwise in Milli-Q water (pH 7.12, 18.2 Ω, recorded at 24 °C). The data shown in this figure has been averaged from 4 scans and smoothed.

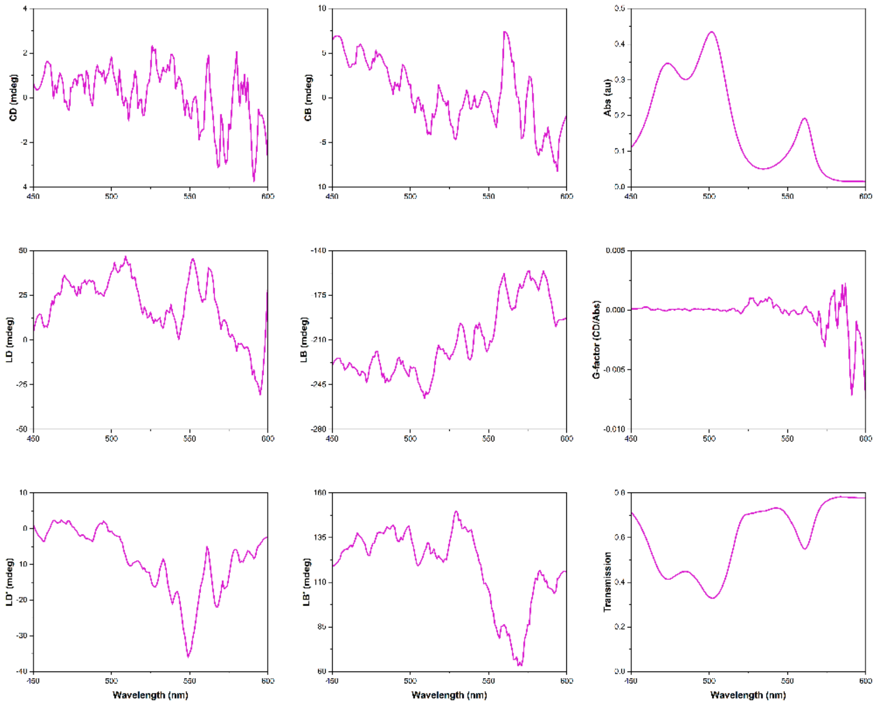

Figure S13: J-aggregates scanned on the MMP formed from non-stirred 25 μM solution of S0271 in Milli-Q water (pH 7.12, 18.2 Ω, recorded at 24 °C). The data shown in this figure has been averaged from 4 scans and smoothed.

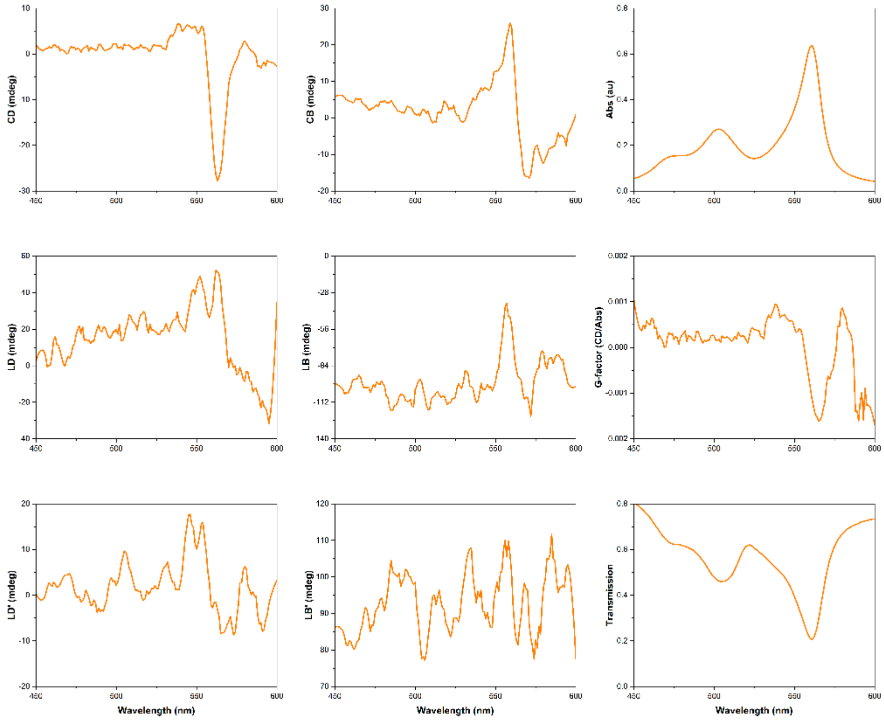

Figure S14: J-aggregates scanned on the MMP formed from stirred a 15 μM solution of S0271 clockwise in Milli-Q water (pH 7.12, 18.2 Ω, recorded at 24 °C). The data shown in this figure has been averaged from 4 scans and smoothed.

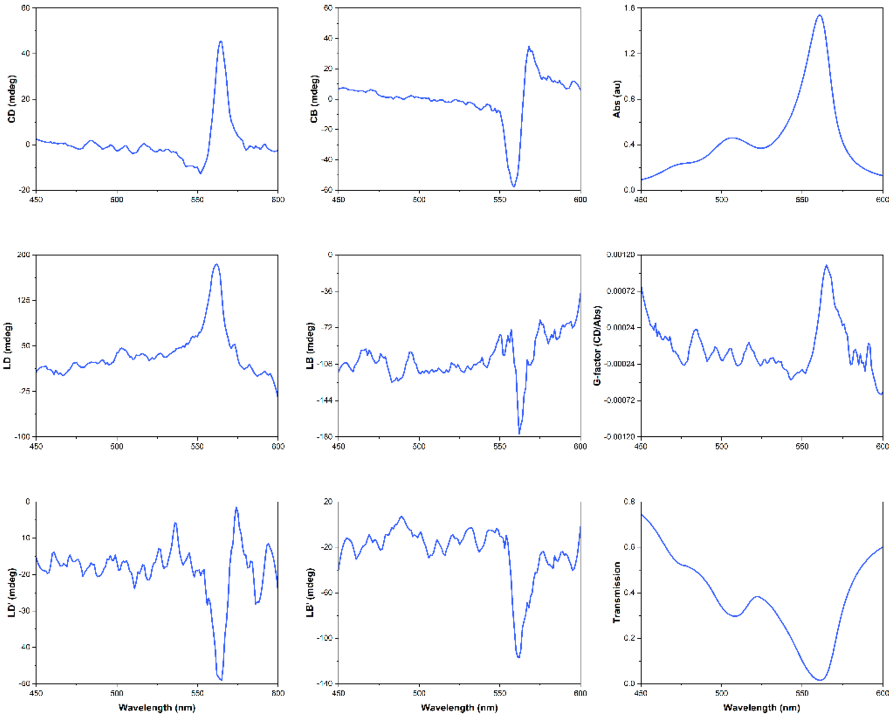

Figure S15: J-aggregates scanned on the MMP formed from stirred a 15 μM solution of S0271 counterclockwise in Milli-Q water (pH 7.12, 18.2 Ω, recorded at 24 °C). The data shown in this figure has been averaged from 4 scans and smoothed.

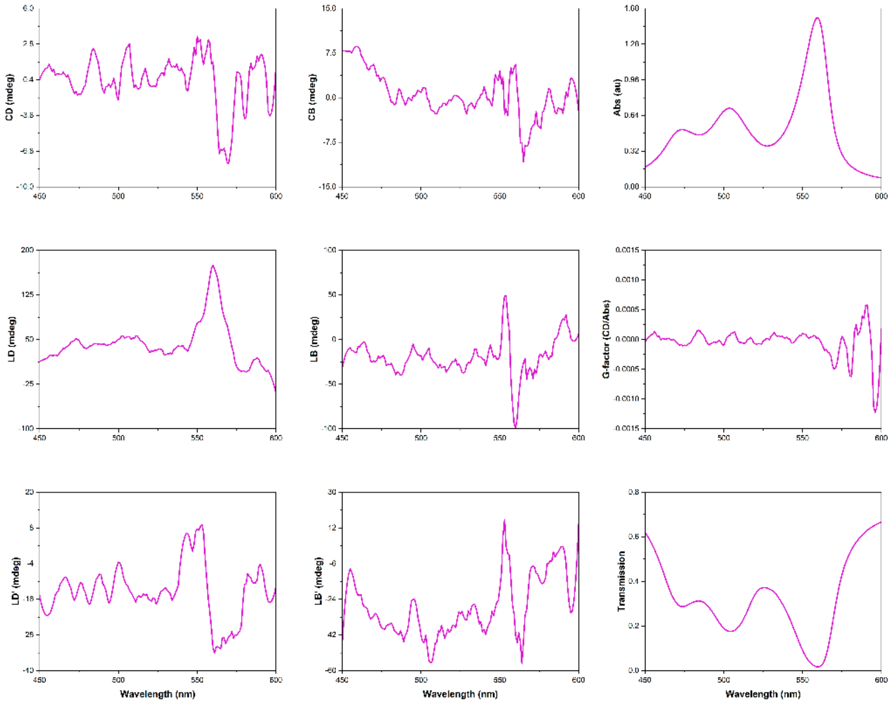

Figure S16: J-aggregates scanned on the MMP formed from non-stirred 15 μM solution of S0271 in Milli-Q water (pH 7.12, 18.2 Ω, recorded at 24 °C). The data shown in this figure has been averaged from 4 scans and smoothed.

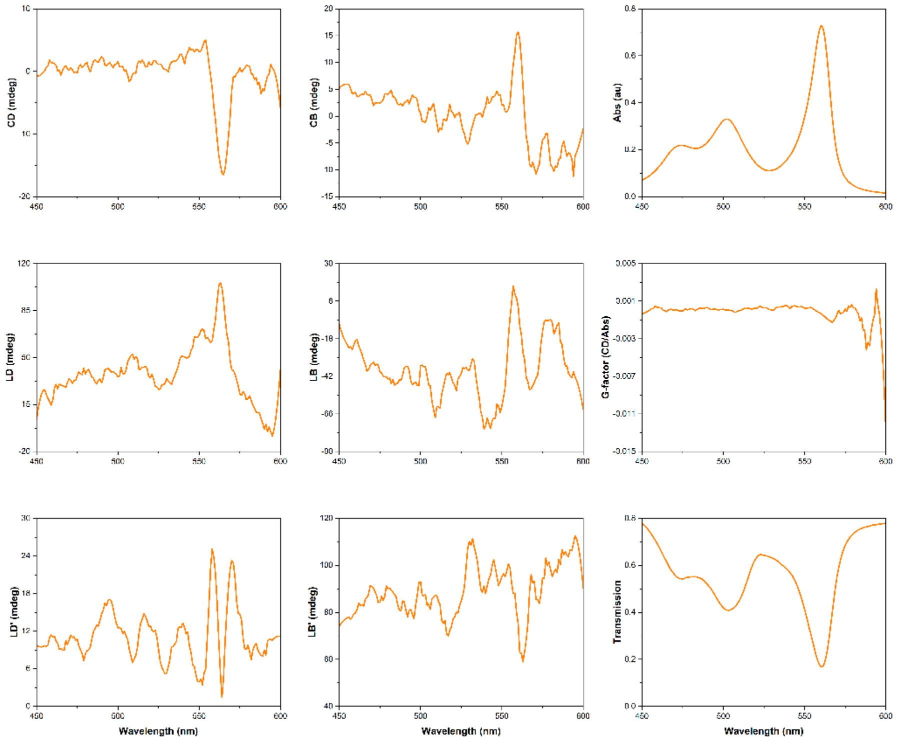

Figure S17: J-aggregates scanned on the MMP formed from stirred a 35 μM solution of S0271 clockwise in Milli-Q water (pH 7.12, 18.2 Ω, recorded at 24 °C). The data shown in this figure has been averaged from 4 scans and smoothed.

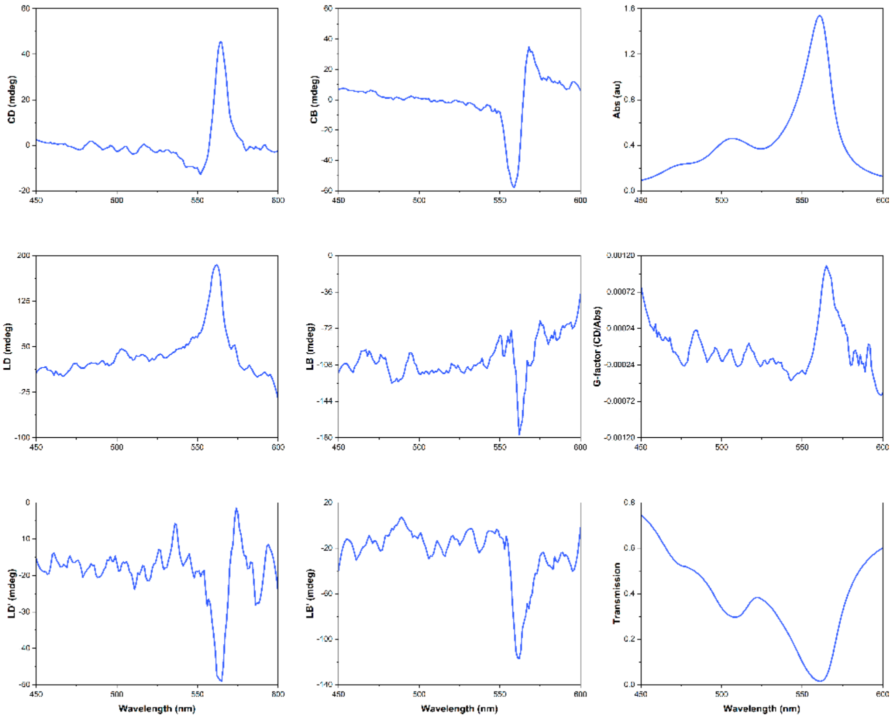

Figure S18: J-aggregates scanned on the MMP formed from stirred a 35 μM solution of S0271 counterclockwise in Milli-Q water (pH 7.12, 18.2 Ω, recorded at 24 °C). The data shown in this figure has been averaged from 4 scans and smoothed.

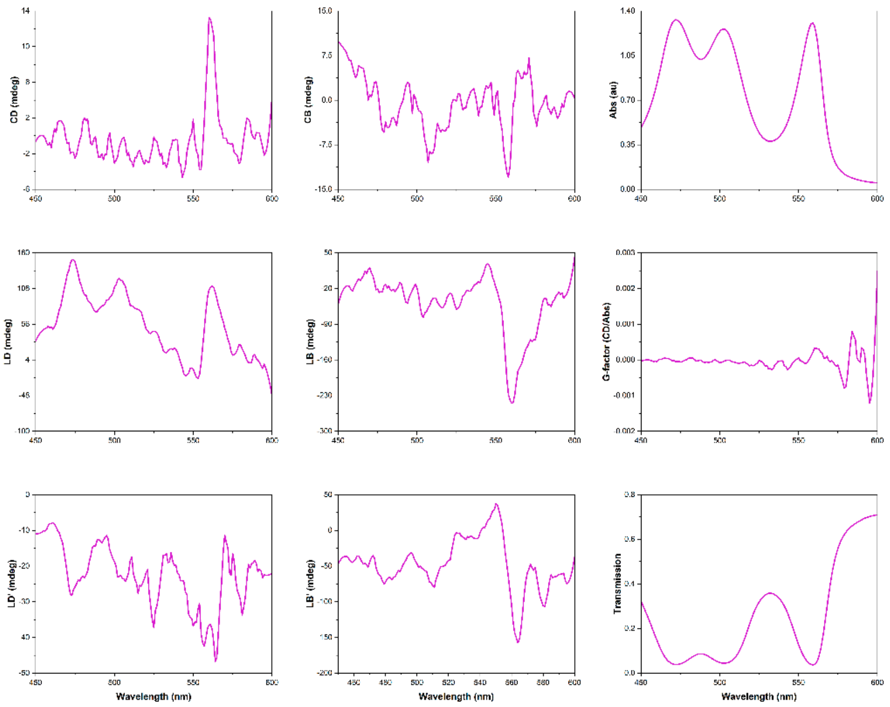

Figure S19: J-aggregates scanned on the MMP formed from non-stirred 35 μM solution of S0271 in Milli-Q water (pH 7.12, 18.2 Ω, recorded at 24 °C). The data shown in this figure has been averaged from 4 scans and smoothed.

4. Combined Normalised via Z-scores Mueller Plots

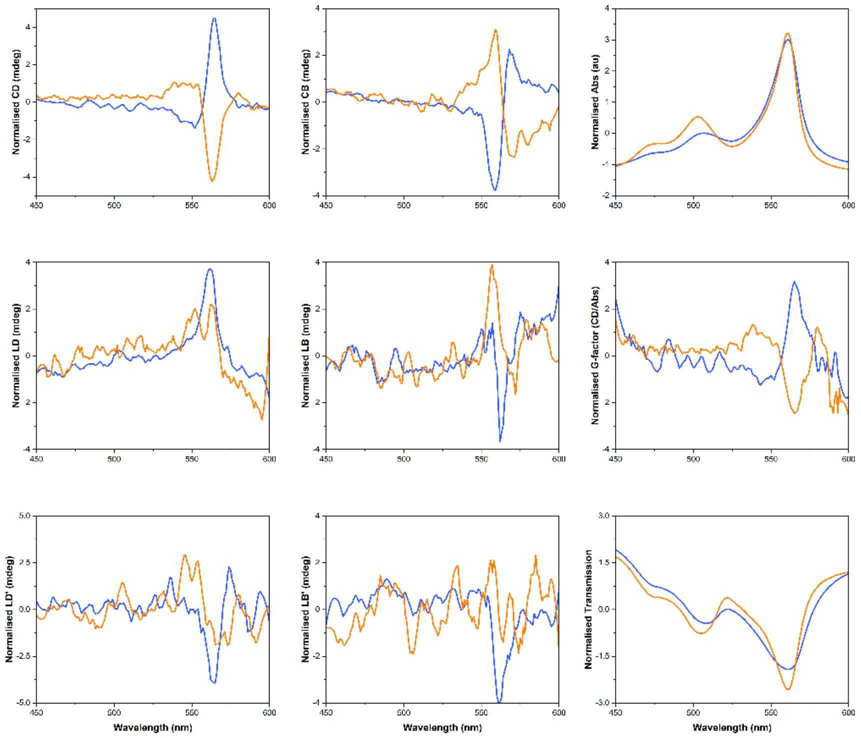

Figure S20: Normalised MMP plots (through Z-scores) of J-aggregates formed from stirring 15 μM solutions of S0271 in Milli-Q water (pH 7.12, 18.2 Ω, recorded at 24 °C) in opposing directions of CW (orange) and CCW (blue).

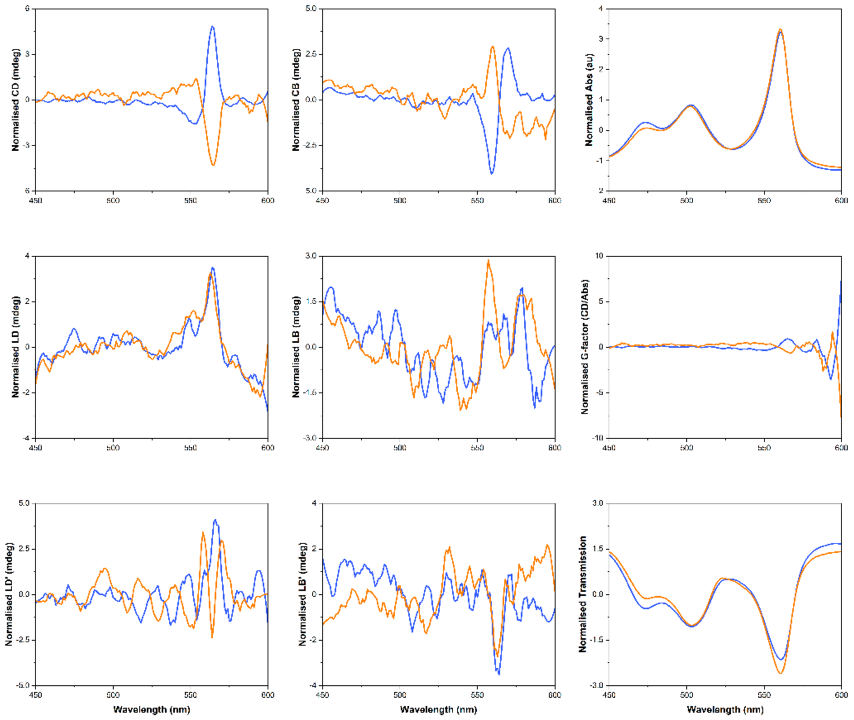

Figure S21: Normalised MMP plots (through Z-scores) of J-aggregates formed from stirring 35 μM solutions of S0271 in Milli-Q water (pH 7.12, 18.2 Ω, recorded at 24 °C) in opposing directions of CW (orange) and CCW (blue).

5. Mueller mapping plots of J-aggregate Films

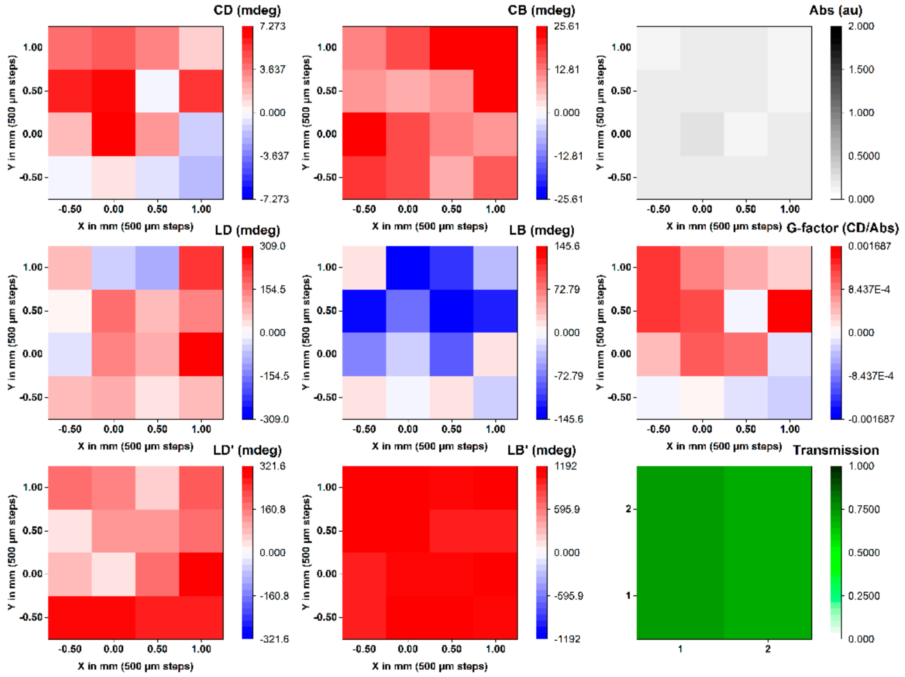

Figure S22: MMP map of a previously CCW stirred 15 μM solution of S0271 in Milli-Q water (pH 7.12, 18.2 Ω, recorded at 24 °C) made *via* dropcasting.

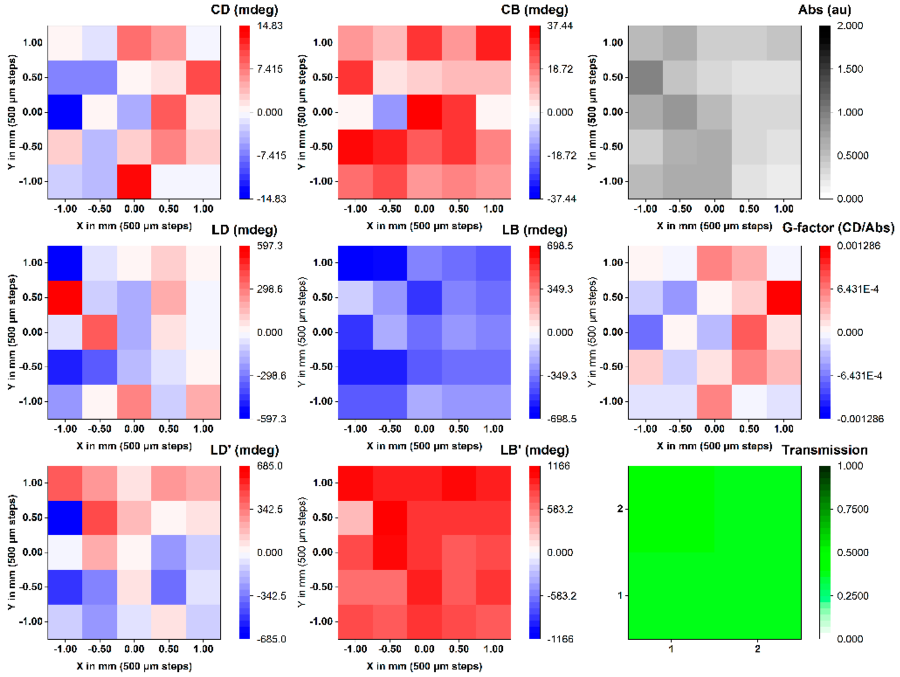

Figure S23: MMP maps of a previously CW stirred 35 μM solution of S0271 in Milli-Q water (pH 7.12, 18.2 Ω, recorded at 24 °C) made *via* dropcasting. The calculated CD, CB, Abs, LD, LD g-factor (CD/Abs), LD′, LB′ maps of the 5×5 grid corresponding to 25 data points measured at 562 nm. These maps were calculated using the Matrix Logarithm method of the differential Mueller matrix measurements [23] and the average of the main diagonal elements (Transmission) of the differential Mueller 4x4 matrix.
